# 3D Point Cloud Object Detection Method Based on Multi-Scale Dynamic Sparse Voxelization

**DOI:** 10.3390/s24061804

**Published:** 2024-03-11

**Authors:** Jiayu Wang, Ye Liu, Yongjian Zhu, Dong Wang, Yu Zhang

**Affiliations:** 1 School of Computer Science and Information Technology, Shanghai Institute of Technology, Shanghai 200235, China; 216142137@mail.sit.edu.cn (J.W.); ly@sit.edu.cn (Y.L.); yuzhang@sit.edu.cn (Y.Z.); 2 The College of Engineering Physics, Shenzhen Technology University, Shenzhen 518118, China

**Keywords:** point cloud, 3D object detection, autonomous driving, convolutional neural networks

## Abstract

Perception plays a crucial role in ensuring the safety and reliability of autonomous driving systems. However, the recognition and localization of small objects in complex scenarios still pose challenges. In this paper, we propose a point cloud object detection method based on dynamic sparse voxelization to enhance the detection performance of small objects. This method employs a specialized point cloud encoding network to learn and generate pseudo-images from point cloud features. The feature extraction part uses sliding windows and transformer-based methods. Furthermore, multi-scale feature fusion is performed to enhance the granularity of small object information. In this experiment, the term “small object” refers to objects such as cyclists and pedestrians, which have fewer pixels compared to vehicles with more pixels, as well as objects of poorer quality in terms of detection. The experimental results demonstrate that, compared to the PointPillars algorithm and other related algorithms on the KITTI public dataset, the proposed algorithm exhibits improved detection accuracy for cyclist and pedestrian target objects. In particular, there is notable improvement in the detection accuracy of objects in the moderate and hard quality categories, with an overall average increase in accuracy of about 5%.

## 1. Introduction

In the context of autonomous driving, the detection, recognition, and localization of key targets have always been one of the most critical tasks. With the development of sensor devices, computing facilities, and deep learning techniques, various algorithms for target detection have been continuously updated and iterated, leading to significant improvements in real-time performance and accuracy. This has also resulted in breakthroughs in the application of target detection technology. In practical autonomous driving scenarios, perception is an important module within the autonomous driving system, and the information obtained from perception guides downstream control systems to generate corresponding control signals [1]. The data sources for perception can be roughly divided into two parts: image information and point cloud information. Unlike image information, point cloud information contains rich 3D data and possesses certain resistance to interference, thus having greater advantages in specific situations. Therefore, point cloud perception has been receiving increasing attention. Point cloud-based detection methods perform well in detecting larger objects [2]. However, due to the uncertainty of the shape of small objects during data collection and the sparsity and disorderliness of point cloud data, there are still significant challenges in detecting small objects.

Point cloud-based object detection emerged after the widespread application of deep learning. Traditional methods struggle to effectively describe and extract features from point cloud data due to their discrete and sparse nature, resulting in traditional point cloud processing methods being limited to data preprocessing stages such as denoising and filtering. The powerful learning capability of neural networks allows the potential of 3D point clouds to be fully utilized.

The PointNet network proposed by Qi et al. [3]. was the first algorithm capable of directly processing irregular point cloud data. This method treats point clouds as unordered sets of points, avoiding complex preprocessing procedures and enabling the learning of local information from point clouds at different scales. However, due to computational resource requirements and limitations on local information about point clouds, this method can only handle small-scale point cloud data. Nevertheless, it provides a solid foundation for subsequent tasks such as classification and segmentation based on point cloud data. To process point cloud data, the problem of disorder needs to be addressed. Voxel-based methods [4] are currently popular point cloud detection methods. These methods primarily transform point cloud data into a collection of three-dimensional voxels, dividing them into equidistant 3D voxels to solve the problem of disorder. Subsequently, object detection is performed through steps such as feature extraction on each voxel. However, due to the use of 3D convolutions in feature extraction and the random sparsity of points within voxels, substantial computational and storage resources are consumed. Sparse convolutional networks are neural networks used to process sparse data. Compared to 3D convolutions, sparse convolutional networks can better handle irregular data by dynamically allocating the number and size of convolutional kernels based on the sparsity, density, and spatial distribution of the input data. They significantly improve computational efficiency and are generally used in combination with voxel-based methods. PointPillars processes point clouds into pseudo-images using a network [5], allowing mature 2D detection methods to be employed in subsequent detection processes. By utilizing 2D convolutions, the overall detection speed and accuracy are improved. This method performs well in detecting large objects such as vehicles but still has room for improvement in detecting small objects like bicycles and pedestrians.

### 1.1. Challenges

Based on the preceding introduction, it is evident that point cloud target detection encounters several challenges.

Primarily, the challenge stems from the nature of point cloud data. The accurate detection of small objects remains challenging in scenarios such as automatic driving, where high precision is essential. This challenge arises from the uncertain shape of small objects as well as the sparse and disorderly nature of point cloud data during data acquisition. Additionally, the discrete and sparse nature of point cloud data poses difficulties in adequately describing and extracting features using a singular approach. Consequently, challenges persist in the extraction and comprehension of complex features.

Secondly, a challenge arises from the limitations of current methodologies. Certain advanced methods for point cloud target detection may demand substantial computing resources, thereby potentially impeding practical implementation. Moreover, early methods often fail to account for both local and global features, resulting in diminished performance when confronted with intricate scenes or small objects.

Furthermore, striking a balance between speed and accuracy poses a challenge as some methods prioritize detection speed over accuracy, potentially compromising detection precision.

### 1.2. Purpose of the Study

To address these issues, this paper proposes a three-dimensional point cloud object detection method based on dynamic sparse voxels and presents the design of a multi-scale feature [6] fusion module to enhance the detection accuracy of small objects in terms of feature extraction, learning, and fusion. The small objects in this paper refer to relatively difficult-to-detect targets, including those with poor quality and fewer pixels, especially targets such as bicycles and pedestrians with less information compared to vehicles. Firstly, by employing dynamic sparse window attention, sparse 3D voxels are processed in parallel to obtain spatial information and 3D features of point cloud data. This step effectively extracts global information and long-distance features from point clouds. In the subsequent feature fusion module, multi-scale features are aggregated and linked to obtain more detailed feature information from point clouds, thereby improving the overall detection accuracy of the model.

## 2. Related Work

### 2.1. Raw Point-Based Method

Point-based methods refer to methods directly operating on point clouds, allowing for the most comprehensive and rich information to be obtained from the data. Due to the discrete nature of point clouds, other methods tend to suffer from varying degrees of information loss. PointNet [3] was the first method to achieve object detection by directly processing point clouds. It has shown good performance in tasks such as object detection and semantic segmentation. However, this network has high computational requirements during data processing, making it unable to meet the demands of large-scale point cloud feature extraction.

Subsequently improved versions, such as PointNet++ [7] and PointRCNN [8], have further enhanced the detection performance based on PointNet. The former reduces the scale of point clouds through sampling and then utilizes PointNet for feature extraction. The latter is a two-stage network model that generates a small number of bounding box proposals in the first stage to facilitate better learning of local point cloud information in the subsequent stages. The original point-based methods generally face problems such as a large amount of data computation, insufficient feature extraction, and loss of implicit feature information.

Point-based methods generally suffer from issues such as high computational complexity and insufficient global feature information, which limit their ability to meet the requirements of complex scenes and practical applications.

### 2.2. Voxel-Based Method

Voxel-based object detection methods refer to the conversion of point cloud data into voxel grid representations and using 3D convolutional neural networks (CNNs) to process the voxel grids for the recognition and localization of 3D objects. VoxelNet [4] converts point clouds into voxel grid representations and employs 3D CNNs to process the voxel grids, modeling point clouds in a dense voxel representation. It extracts object features through convolutional operations. However, the inclusion of empty voxels resulting from the neglect of point cloud sparsity leads to storage space and computational resource wastage, limiting the network’s performance. TANet [9] attempts to use attention mechanisms to enhance discriminative points and suppress unstable points to solve the problem, achieving certain results. However, the effect is still average for large-scale sparse point clouds.

Yan et al. [10]. proposed a method called SECOND, which effectively learns sparse voxel level features from point clouds using sparse convolution. PointPillars [5] transforms point clouds into pseudo-images and utilizes 2D CNNs to extract features, significantly enhancing detection speed. Recently, the introduction of attention mechanisms [11] and the utilization of sparse convolution have greatly facilitated the development and usage of voxel-based methods.

The performance and computational speed of voxel-based methods are impressive, but due to the sparse nature of point clouds, some network performance is limited, and new methods need to be introduced to solve this problem.

### 2.3. Sparse Convolution Methods

As mentioned earlier, the sparsity of point clouds hinders the performance of traditional convolutional neural networks. Unlike 2D images, most 3D point clouds have empty voxels, which waste storage space and computational resources. To effectively calculate the convolution of sparse data in the raw point cloud, rather than scanning all spatial voxels, the sparse convolution method is proposed. The submanifold sparse convolutional network [12,13] commonly used in point cloud processing reduces computational complexity by establishing a position hash table and RuleBook on non-empty data and only computing convolution on valid data.

### 2.4. Contribution

We propose a solution to address issues with the existing methods mentioned above. We made modifications based on PointPillars. We improved the original network by incorporating a novel 3D feature extraction module, DSV [14], which can better extract global information and long-range features from point clouds. Through a window-based attention strategy, this method can effectively parallel process sparse 3D voxels while improving the efficiency of obtaining features for small objects. This module serves as the backbone network to enhance the network’s feature extraction capability.

We introduced a multi-scale feature fusion module to fuse information from small object detection and features at different scales, resulting in a feature map that contains more information.

We evaluated the performance of the network using the KITTI benchmark and improved the detection accuracy of cars, bicycles, and pedestrians without sacrificing speed, especially for bicycles and pedestrians.

In Table 1, we have summarized the methods mentioned earlier to provide a complete perspective on previous research.

## 3. Model Structure

**Figure 1:**
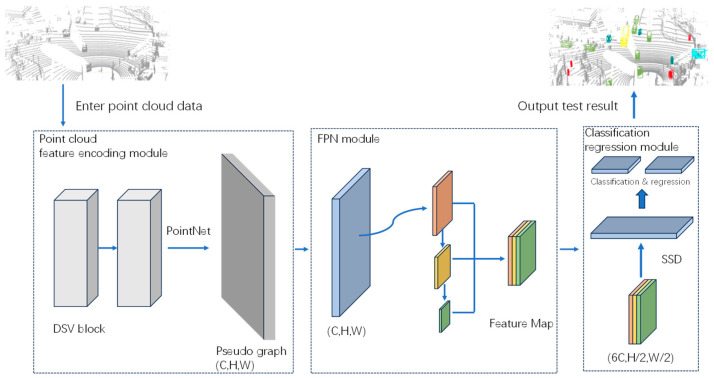
Structure of the 3D point cloud object detection method based on dynamic sparse voxelization.

The structure of the proposed dynamic sparse voxelated 3D point cloud object detection method is illustrated in Figure 1. It consists of a feature encoding module utilizing DSV blocks, a feature fusion FPN module, and a detection classification regression module. In addressing the need to improve the detection of small objects, the DSV module is capable of better extracting global features and long-distance feature information from the point cloud, thereby enabling improved aggregation of shallow small object information. Experimental validation confirmed the favorable detection performance of the proposed method on the public KITTI dataset.

### 3.1. Dynamic Sparse Voxel Transformer Block

The transformer is a type of deep learning model that heavily relies on the self-attention mechanism to model long-range dependencies within sequences. Existing point cloud processing methods often focus only on local features, while the introduction of the transformer enables the management of arbitrarily sized sparse voxels over a large range, leading to improved learning effectiveness for voxels with similar features across the global scope. Due to the sparsity of 3D point clouds, many voxels do not contain points, and the number of points in non-empty voxels varies widely. Directly applying standard transformers is challenging, as padding empty regions involves computationally intensive operations. Therefore, the introduction of this method allows for parallel processing of sparse 3D voxels.

After converting the point cloud into voxels, the voxels are further divided into a series of non-overlapping windows, each containing N non-empty voxels (with different N values for each window). Within the current window, each voxel can be represented by Equation (1), including the spatial coordinates ($x_{i}$, $y_{i}$, and $z_{i}$) of the voxel within the window, the corresponding feature map $f_{i}$, and an inner window voxel ID $d_{i}$. Here, $d_{i}$ is a sequential number assigned to the voxel within the window, independent of spatial coordinates, that depends on the subsequent subset division approach. For instance, subsequent division may be carried out along the x and y directions, resulting in different voxel IDs $d_{i}$ assigned to voxels at the same position.

(1) $$v={\left\{\left.v_{i}\right|v_{i}=\left[\left(x_{i}{,y}_{i}{,z}_{i}\right){;f}_{i}{;d}_{i}\right]\right\}}_{i=1}^{N}$$

For each window, it is further partitioned into equitably sized subsets, ensuring that each subset comprises a maximum of τ non-empty voxels (where τ is a hyperparameter). The calculation formula, demonstrated by Equation (2), is then used to determine the value of S for each window based on its individual sparsity. Subsequently, the window is divided into S (N/τ) rounded-down subsets, enabling the allocation of computational resources in proportion to their sizes and achieving dynamic resource allocation.

(2) $$S=\left[\frac{N}{\tau }\right]+\left[\left(N\%\tau \right)>0\right]$$

So far, in the process of obtaining the feature for each voxel, given all voxels V within a window, along with the i-th subset Q and the voxel $d_{i}$ D within this window, the feature map F and their spatial coordinates O can be retrieved for all voxels within this subset through index operations, as demonstrated by Equation (3).

(3) $$F_{j,}O_{j}=\mathrm{INDEX}\left({V,Q}_{j},D\right)$$

To establish connections between different voxels within the same window, an attention mechanism is introduced. This attention mechanism consists of two layers of self-attention, employing different voxel ordering methods to generate different subsets. The first layer sorts voxels along the x-axis direction, while the second layer sorts them along the y-axis direction. Subsequently, a multi-head self-attention layer is utilized to establish connections between different subsets in this manner.

**Figure 2:**
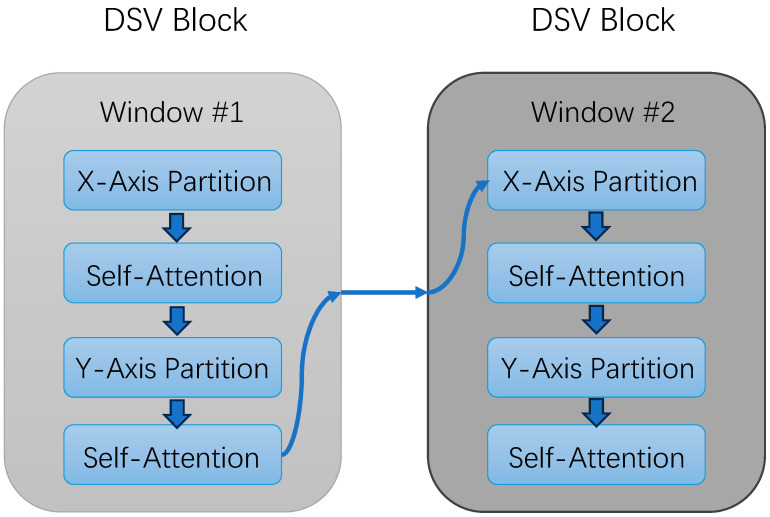
The structure of the DSV block.

To establish connections between voxels across different windows, a sliding window approach, as employed in the swing transformer, is adopted here, enabling the reorganization of windows. Furthermore, the size of windows is subject to change, with adjacent blocks in the network structure employing windows of different sizes, and an internal shift can also be performed. Upon completion of this encoding process, the extracted feature map is provided for subsequent module usage. The structure of the block is illustrated in Figure 2. Through point-pillar scattering, it is transformed into a 2D pseudo-image of size (C, H, W), and a 2D backbone is utilized to extract features once again.

### 3.2. Multi-Scale FPN Module

**Figure 3:**
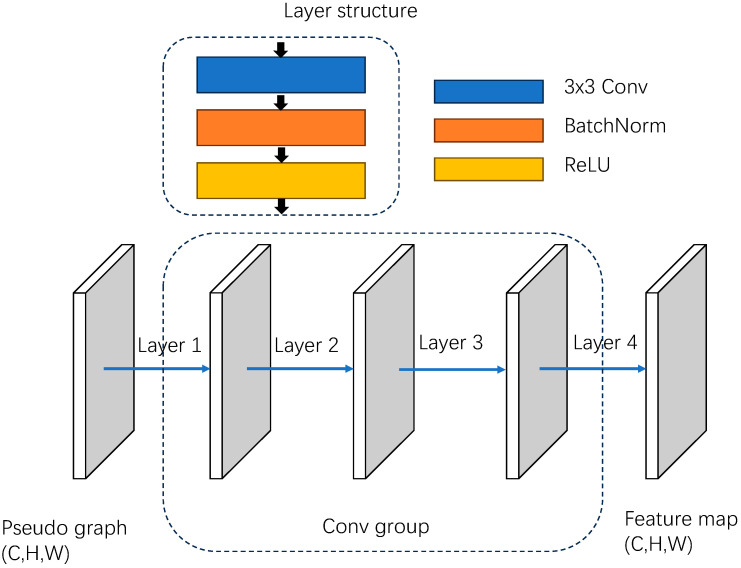
Structure of the convolution group.

Considering that shallow-level feature maps can retain the richest and most accurate information for small targets, feature extraction and aggregation are performed on the pseudo-images using a multi-scale FPN module. The pseudo-images are obtained by spatial cluster convolution to yield feature maps consistent with the input dimensions. The detailed structure is depicted in Figure 3. Each of these layers consists of a 3 × 3 convolutional layer, a normalization layer, and an activation function. By ensuring a certain network depth and employing non-linear activation functions, the unstructured nature of the network is improved. Subsequently, a convolution operation is applied to the input feature map (C, H, W) to yield a feature map of size (2C, H/2, W/2), followed by multi-scale aggregation of the extracted feature maps in the subsequent process.

By sampling the feature maps through multi-scale convolutions, feature maps of dimensions (C, H/2, W/2), (C, H/4, W/4), and (C, H/8, W/8) are obtained. Subsequently, each scale of feature map undergoes deconvolution to yield three feature maps of size (2C, H/2, W/2). Finally, the three processed and sampled feature maps are concatenated to obtain a fused feature of output size (6C, H/2, W/2). Specifically, the convolution operation formula is given by Equation (4).

(4) $$H_{2}=\frac{H_{1}-F+2P}{S}+1$$

In the equation, H_2_ represents the size of the output feature map, and H_1_ represents the size of the input feature map. F represents the size of the convolution kernel, P represents the size of the padding, and S represents the stride size.

Similarly, the operation formula for deconvolution is given by Equation (5).

(5) $$H_{4}=\left(H_{3}-1\right)\cdot S+F-2P$$

where H_4_ denotes the size of the output feature map of deconvolution, and H_3_ denotes the size of the input feature map of deconvolution.

**Figure 4:**
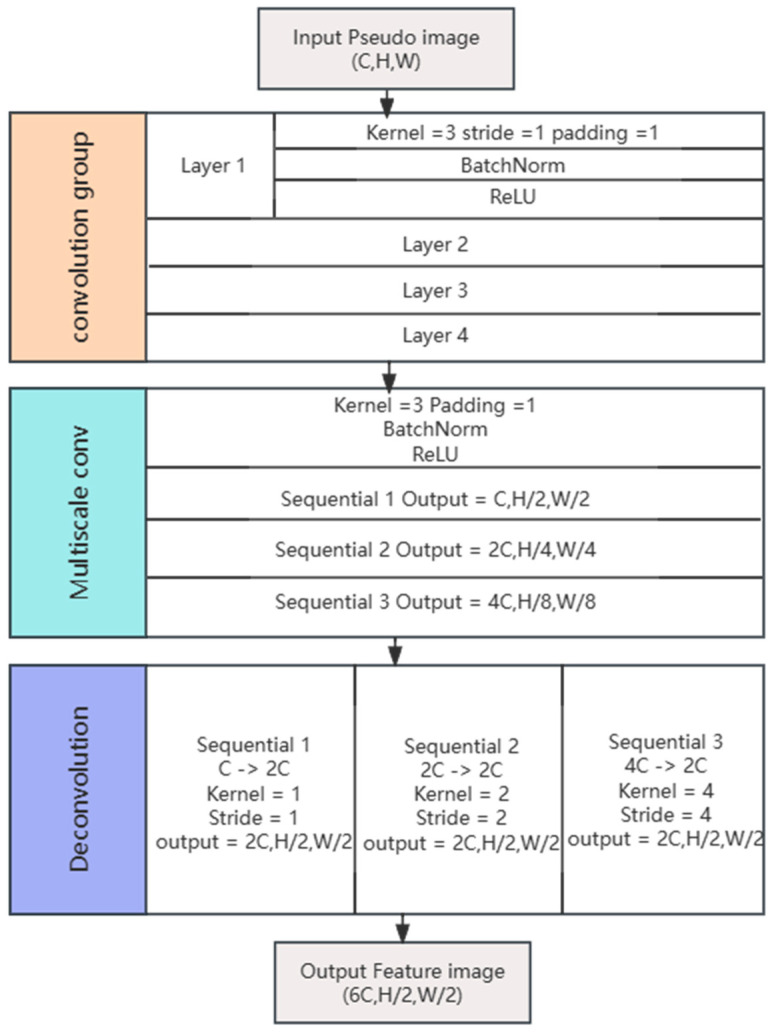
The structure and parameters of the convolutional network.

The structure and partial parameters of the convolutional layer are illustrated in Figure 4. This fused feature is then fed into the SSD [15] for target classification and regression detection.

### 3.3. Classification Regression Module

This module employs a detection head similar to the single-stage detector (SSD) to perform object classification and regression predictions on the fused features (6C, H/2, W/2). To measure the matching degree between 3D bounding boxes and ground truth, Union IoU [16] is introduced for qualitative assessment. The key to 3D object detection lies in accurately classifying and localizing the target objects in 3D space. The loss function used in this study is consistent with PointPillars, Firstly, ground truth and anchor points are defined by (x, y, z, w, l, h). What needs to be focused on in the subsequent tasks is the offsets of these seven variables, and the smoothL1 [17] loss function is employed for bounding box regression. The total regression loss is given by Equation (6).

(6) $$\mathrm{SL}=\sum_{b\in \left(x,y,z,w,l,h,\theta \right)}\mathrm{SmoothL}1\left(\Delta b\right)$$

where x, y, and z are the center coordinates of the target; l, w, and h represent the length, width, and height; and θ is the rotation angle around the z-axis.

Similarly, to avoid orientation ambiguity, the Softmax loss is introduced to learn the direction of objects. This loss is denoted as L_dir_. To address the imbalance issue between foreground and background classes commonly found in point cloud space, the focal loss function proposed in RetinaNet [18] is introduced for multi-object classification loss calculation. This is represented by Equation (7).

(7) $${\mathrm{FL}=-\alpha }_{a}{\left({1-p}^{a}\right)}^{\gamma }{\log p}^{a}$$

Here, p represents the class probability of 3D bounding boxes with the hyperparameters set as α = 0.25 and γ = 2. Finally, the total loss is computed as shown in Equation (8).

(8) $$L=\frac{1}{N_{\mathrm{pos}}}\left(\beta_{\mathrm{sl}}{\mathrm{SL}+\beta }_{\mathrm{fl}}{\mathrm{FL}+\beta }_{\mathrm{dir}}L_{\mathrm{dir}}\right)$$

where N_pos_ is the positive sample aim point and the loss weights are set to (β_sl_ = 1.0, β_fl_ = 2.0, β_dir_ = 0.2).

## 4. Experiment and Result Analysis

### 4.1. Data Set

The KITTI dataset [19] is a widely used public dataset for autonomous driving research and computer vision tasks. It provides various sensor data in real-world scenarios and includes a rich variety of object categories. In this experiment, the main detection targets were cars, cyclists, and pedestrians. The KITTI dataset evaluates detection results using the average precision of both the bird’s-eye view (BEV) and 3D modalities.

The dataset was divided into 7481 training and validation samples as well as 7581 test samples. For different objects, the dataset categorizes the difficulty of 3D object detection based on factors such as size, point cloud density, and occlusion. The difficulty levels are classified as easy, moderate, and hard. An evaluation was conducted based on the detection results of different objects. Higher pixel values indicate lower occlusion and truncation levels, making identification easier, while lower pixel values indicate higher difficulty in recognition.

In this experiment, our focus was on improving the detection accuracy of small targets. Specifically, small targets refer to objects in the utilized dataset that are relatively smaller and have fewer pixels compared to easily recognizable cars. These small targets include cyclist and pedestrian objects that are less easily identifiable due to their lower pixel count. Additionally, they encompass moderate and hard samples in the dataset, which have a limited number of effective pixels (less than 25) and varying degrees of occlusion. The specific classification details and criteria are presented in Table 2 below.

### 4.2. Implementation Details

The experimental hyperparameters were as follows: the batch size was 6, the optimizer used was AdamW, the learning rate was set to 0.001, and the weight decay was set to 0.9. The IoU threshold for cars was set to 0.7, while it was set to 0.5 for pedestrians and cyclists.

Regarding the hardware conditions, the operating system used was Ubuntu 20.04, and there were two NVIDIA RTX 4090 GPUs (ASUS Taiwan, China) in the configuration. The software used for the experiment included Python 3.8, PyTorch 1.8.0, CUDA 11.6, and cuDNN 8.0.3, which were employed to accelerate computations.

**Figure 5:**
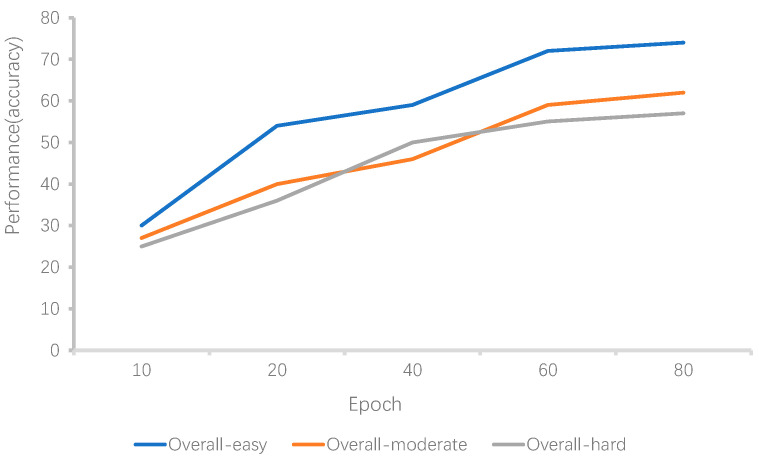
Training process illustration.

The experiment consisted of a total of 80 training epochs, and it took approximately 15 h to complete. During the entire training process, the validation results of the overall samples varied with the change in training epochs, as depicted in Figure 5.

### 4.3. Analysis of Test Results

**Figure 6:**
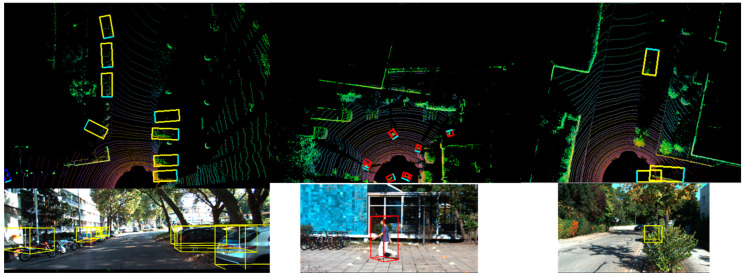
Visual detection results of a point cloud.

To visually demonstrate the detection performance of the algorithm, we used Open3D (0.17.0) to display the detection results. Visualization was achieved through bird’s-eye view rendering and projecting 3D bounding boxes onto the images. The bird’s-eye view was derived from the dataset, where the point cloud space was segmented into voxels. Subsequently, down-sampling of the point cloud was performed using voxels, and each voxel was projected as a point to obtain the projection of the point cloud on the plane perpendicular to the vertical direction. The results are shown in Figure 6. The figure showcases the detection performance for vehicles and pedestrians as well as scenarios involving occlusion. The red boxs represents detected pedestrians, and the yellow boxs represents detected vehicles.

The KITTI dataset was used for training and testing the proposed network, and the average precision (AP) of the bird’s-eye view (BEV) mode and three-dimensional (3D) mode were used to evaluate the detection results on the KITTI benchmark. Table 3 and Table 4 present a performance comparison of our experiment with other point cloud-based object detection algorithms on the KITTI dataset. From the tables, it can be observed that our proposed network architecture exhibited significant advantages in detecting smaller objects such as pedestrians and some bicycles across all difficulty levels.

Compared to the voxel-based PointPillars algorithm, our network structure achieved a minimum of 5% improvement in detection accuracy in both bird’s-eye view and 3D modalities across the three difficulty levels. The evaluation of detection speed was conducted using frames per second (fps) as the metric.

The method we propose is based on improvements to PointPillars, which shows an overall improvement compared to the original method, especially for samples with poor quality such as cyclists and pedestrians, with an improvement of over 3.8% to 5%. We also maintained our original advantages in terms of detection speed as much as possible.

Methods based on the original point cloud, such as PointRCNN, can preserve the richest target information in the input data, thereby achieving better detection accuracy, but the detection speed is often slower. The voxelization method has a fast detection speed but low accuracy. Our proposed method not only improves accuracy but also considers detection speed.

### 4.4. Ablation Experiments

Ablation experiments were conducted to assess the impact of each component of the proposed model on detection accuracy. The roles of the DSV module and the multi-scale FPN module in feature extraction and fusion were examined. The evaluation was performed using the KITTI dataset.

Unified indicators were employed to assess the effect of these two modules on enhancing model detection accuracy. A model excluding these two modules was used as the baseline for testing purposes. Table 5 illustrates that employing solely the DSV module resulted in a 2.68% mAP increase in pedestrian detection over the baseline model, whereas utilizing solely the multi-scale FPN module led to a 1.52% mAP increase compared to the baseline model. In contrast, the combined utilization of both modules resulted in our model achieving superior detection performance, surpassing the baseline model by 4.99% in mAP. Notably, both of our proposed modules exhibited performance enhancements, suggesting that feature extraction by the DSV module and feature fusion facilitated by the multi-scale FPN module are beneficial for small object detection.

## 5. Discussion

Among the existing detection methods, the original point cloud-based method exhibits relatively high accuracy but poor real-time performance, limiting its applicability in complex scenes or autonomous driving environments. On the other hand, the voxel-based method offers fast detection speed but lacks accuracy, particularly for small objects with high detection difficulty.

The method proposed in this paper relies on voxel-based networks, retaining the characteristic of rapid detection speed. Furthermore, the DSV module exhibits a strong feature extraction effect, enhancing the extraction of global features and refining feature granularity. Additionally, the FPN module integrates features of varying scales to acquire more comprehensive feature maps, thereby improving the detection performance of targets of diverse sizes, particularly smaller ones. It achieved an FPS of 45, significantly surpassing that of the point cloud-based method. Simultaneously, the detection accuracy of this method surpassed that of the baseline method by a considerable margin, exhibiting varying degrees of improvement across different object types, particularly in the context of pedestrians, cyclists, and objects with high detection difficulty. Ablation experiments demonstrated the optimization effects of our modules on baseline methods, with the combined effect proving to be the most effective.

Overall, the proposed method presents distinct advantages.

## 6. Limitations and Future Work

The proposed method demonstrates promising results but also presents limitations that we intend to address in our forthcoming research.

The primary concern revolves around generalization performance. While our method significantly improved the detection of pedestrians, bicycles, and other specific targets, additional validation is required for its generalization performance across diverse target types and complex scenarios.

Moreover, in the domain of autonomous driving, the existence of various small object obstacles highlights the necessity to enhance the algorithm’s generalization for diverse target types and complex environments. Secondly, it is essential to evaluate the robustness of our enhanced method under varying environmental conditions, especially in challenging scenarios such as fluctuations in lighting and weather. Subsequent investigations will delve into methods to enhance the algorithm’s robustness to ensure stability and reliability in practical applications.

Lastly, the allocation of computing resources across different hardware platforms remains ambiguous. To improve practical applicability, future considerations will involve exploring optimization algorithms aimed at reducing computing resource demands on varied hardware platforms.

## 7. Conclusions

In response to the shortcomings of existing deep learning networks based on voxel-based methods for point cloud object detection, we devised a method involving dynamic sparse voxelization and fusion modules. This approach combines a point cloud encoding module with a classification–regression–detection head to achieve multi-scale point cloud object detection.

By extracting richer feature information at a finer granularity and incorporating multi-scale features, we significantly improved the accuracy of small object detection while minimizing the loss of fps and ensuring the accuracy of large object detection. The algorithm’s performance was evaluated on the KITTI dataset and compared with other methods, confirming its effectiveness. In the future, we will delve deeper into exploring methods to enhance the accuracy of object detection speed in point clouds.

## Figures and Tables

**Table 1:** Overview of Related Research.

Method	Type	Reference	Dataset	Key Aspects	Limitations
PointNet	Point-based	CVPR 2017	ModelNet40	Provides an efficient method for directly processing point clouds	As a basic method, it usually needs a lot of calculation and can only extract local features
PointRCNN	Point-based	CVPR 2019	KITTI	3D detection method based on the original point cloud	High precision and low speed
VoxelNet	Voxel-based	CVPR 2018	KITTI	Voxel-based 3D detection model	Empty voxels waste computing resources
TANet	Voxel-based	AAAI 2020	AVA/TAD66K	Multiple attention mechanisms	Poor performance of large-scale point clouds
SECOND	Voxel-based	Sensors 2018	KITTI	Sparse convolution	Detection accuracy needs to be improved
PointPillars	Voxel-based	CVPR 2019	KITTI	Pseudo-image	Detection accuracy needs to be improved
Proposed method	Voxel-based	-	KITTI	DSV module, multi-scale FPN module	Generalization, robustness, etc.

**Table 2:** Definition of KITTI dataset detection difficulty.

Grade	2D Detection Box High/Pixel	Occlusion Degree	Degree of Truncation/%
Easy	40	Not covered	<15
Moderate	25	Partial occlusion	<30
Hard	25	Complete occlusion	<50

**Table 3:** Comparison of detection accuracy (BEV) of different algorithms on the KITTI dataset.

Method	Car			Cyclist			Pedestrian			Speed (fps)
SECOND	Easy	Moderate	Hard	Easy	Moderate	Hard	Easy	Moderate	Hard	20
	88.07	79.37	77.95	73.67	56.04	48.78	55.10	46.27	44.76	
VoxelNet	89.35	79.26	77.39	66.70	54.76	50.55	46.13	40.74	38.11	4.4
PointRCNN	92.13	87.93	82.72	82.56	67.24	60.28	54.77	46.13	42.84	10
PointPillars	88.35	86.10	79.83	79.14	62.25	56.00	58.66	50.23	47.19	62
Ours	89.00	86.54	79.61	80.10	64.31	59.81	66.21	56.10	53.73	45
Improvement	+0.65	+0.44	-0.22	+0.96	+2.06	+3.81	+7.55	+5.87	+6.54	-

Specific improvement values are referenced with PointPillars as a benchmark.

**Table 4:** Comparison of detection accuracy (3D) of different algorithms on the KITTI dataset.

Method	Car			Cyclist			Pedestrian			Speed (fps)
SECOND	Easy	Moderate	Hard	Easy	Moderate	Hard	Easy	Moderate	Hard	20
	83.13	73.66	66.20	70.51	53.85	46.90	51.07	42.56	37.29	
VoxelNet	77.47	65.11	57.73	61.22	48.46	44.37	39.48	33.69	31.50	4.4
PointRCNN	86.96	75.64	70.70	74.69	58.82	52.53	47.98	39.37	36.01	10
PointPillars	79.05	74.99	68.30	75.78	59.07	52.92	52.08	43.57	41.49	62
Ours	83.91	76.20	71.58	76.91	61.22	56.79	59.10	51.46	47.53	45
Improvement	+4.86	+1.21	+3.28	+1.13	+2.15	+3.87	+7.02	+7.89	+6.04	-

Specific improvement values are referenced with PointPillars as a benchmark.

**Table 5:** Ablation study of individual components in each module.

DSV	M-FPN	3D Object Detection AP			mAP
		Easy	Mod.	Hard	
×	×	60.66	52.23	48.19	53.69
√	×	63.25	54.01	51.85	56.37
×	√	62.74	53.36	49.53	55.21
√	√	66.21	56.10	53.73	58.68

The average accuracy of BEV for pedestrian detection on the KITTI test set.

## Data Availability

The data used in this paper were obtained from a third-party database (https://www.cvlibs.net/datasets/kitti), accessed on 10 September 2023.

## Abbreviations

The following abbreviations and mathematical variables were used in this paper:

CNN	convolutional neural networks
FPN	feature pyramid network
DSV	dynamic sparse voxelization
BEV	bird’s-eye view
(x, y, z)	spatial coordinates of the voxels
$f_{\;}$	corresponding feature map
$d_{\;}$	inner window voxel ID
N	total voxel window
O	spatial coordinates of all voxels within a subset
V	all voxels within a given window
Q, D	specified subset and voxel
H	size of the feature map
F	size of the convolution kernels
P	size of the padding
S	size of the step
(w, l, h, θ)	length, width, height, and the rotation angle around the z-axis
L_dir_	object directional loss
FL	multi-object classification loss
SL	bounding box regression loss
L	total loss
